# Controlled Lipid Domain Positioning and Polarization in Confined Minimal Cell Models

**DOI:** 10.1002/anie.202419529

**Published:** 2025-01-07

**Authors:** Koyomi Nakazawa, Antoine Lévrier, Sergii Rudiuk, Ayako Yamada, Mathieu Morel, Damien Baigl

**Affiliations:** ^1^ PASTEUR Department of Chemistry École Normale Supérieure PSL University Sorbonne Université CNRS 75005 Paris France

**Keywords:** Synthetic Cell, Polarization, Lipid domain, Microfluidics, Giant Unilamellar Vesicle

## Abstract

Giant unilamellar vesicles (GUVs) are widely used minimal cell models where essential biological features can be reproduced, isolated and studied. Although precise spatio‐temporal distribution of membrane domains is a process of crucial importance in living cells, it is still highly challenging to generate anisotropic GUVs with domains at user‐defined positions. Here we describe a novel and robust method to control the spatial position of lipid domains of liquid‐ordered (Lo)/liquid‐disordered (Ld) phase in giant unilamellar vesicles. Our strategy consists in confining Lo/Ld phase‐separating GUVs in microfluidic channels to define free curved regions where the minority‐phase domains localize and coalesce by decreasing the line energy through domain fusion. We show that this process is governed by the respective fraction of the two phases, and not by the chemical nature of the lipids involved. The spatial position and number of domains are controlled by the design of the confining microchannel and could result in polarized GUVs with a controllable number of poles. The developed method is versatile and user‐friendly, while allowing multiple single‐vesicle experiments in parallel.

## Graphical abstract

Microfluidic confinement of phase‐separating giant unilamellar vesicles (GUVs) directs the accumulation of the minority‐phase domains in the free regions where they fuse to minimize their line energy. This results in the localization of lipid domains at prescribed positions and the possibility to build polarized cell models with a control over the number and position of the poles.

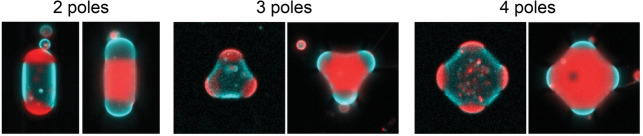

## Introduction

Living cells are characterized by a well‐defined and highly‐regulated spatiotemporal organization of their biomolecular content. In particular, non‐uniformity in molecules concentration (gradients) and distribution (e.g., polarization), is capital in many key biological processes, from apical‐basal establishment in epithelial cells^[1]^ to asymmetric division in stem cells,^[2]^ or directional sensing and motility in neuronal growth cones.^[3]^ In bacteria, polarization has also a pivotal role in crucial processes such as cell division, virulence, chemotaxis, motility and adhesion.^[4]^ For most of these phenomena, the spatial organization of membrane proteins and associated lipids, constituting the interface with the surrounding micro‐environment, contribute to the specificity of the signaling outcome.^[5]^ Mechanisms driving the spatial organization of the lipid membrane are thus of particular importance to understand biological polarization.^[6]^ For example, microdomains of ordered lipid phase, called “lipid rafts”, are hypothesized to recruit specific sets of transmembrane and scaffolding proteins for functional processes.^[7]^ However, the understanding of the underlying mechanisms directing ordered/disordered lipid phase segregation as well as further recruitment of specific lipids and proteins in sub‐domains, remains incomplete in particular due to the difficulty of extracting essential parameters in the highly crowded and multi‐factorial environment of living cells.

There is thus a need for the development of reconstituted *in vitro* systems, in which the different components and physico‐chemical parameters could be finely tuned and precisely ordered in space and time. To this aim, giant unilamellar vesicles (GUVs), which are spherical entities with a phospholipid bilayer membrane and a diameter (1–100 μm) comparable to that of actual cells, appear as particularly suitable minimal models to mimic living cells.^[8]^ Various methods have been developed to control their size, inner content and composition of their membrane (in lipids and proteins), leading to various relevant examples of synthetic cells, each of them mimicking, reproducing, or allowing to study one or several specific biological features.[^8^, ^9^, ^10^] Hence, the formation and organization of lipid domains have been studied using GUVs as models for lipid phase segregation.[^11^, ^12^, ^13^] Indeed, mixtures of unsaturated and saturated lipids with cholesterol in proper proportions exhibit liquid‐ordered (Lo)/liquid‐disordered (Ld) phase separation.^[13]^ Controlling domain formation in GUVs, either by lipid stoichiometry^[13]^ or externally by temperature^[13]^ or light stimulus,^[14]^ is a well‐established process, but the control over the domain spatial distribution remains a highly desired challenge, which has been partially achieved in only a few notable reports. For instance, microstructured substrates have been shown to direct the position of lipid domains at specific locations but this involved the use of supported bilayers[^15^, ^16^] and not GUVs. To our knowledge, spatial control over lipid domain position in GUVs has been only realized either on a single vesicle under pneumatic stress^[17]^ or on GUVs squeezed in pH‐sensitive gels.^[18]^ The first study showed that, in a GUV compressed into a pancake shape between two walls, the lipid phase in minority tended to localize away from the flat parts in contact with the walls. This approach was however limited to a single compressed GUV and did not allow much flexibility in terms of domain localization. In contrast, the second study allowed to impose a broad variety of user‐defined shapes to a large number of constrained GUVs, but it required a specific pH‐sensitive gel microenvironment and the control over domain distribution was not studied in detail.

Here, we report a systematic method investigating how domains of interest can be localized at prescribed positions in many GUVs in a fluid environment. Our strategy consisted in confining vesicles in microfluidic channels to create free regions of curved membrane where the lipid phase in minority accumulated and formed a single domain minimizing line energy. GUVs of different Lo/Ld compositions were first confined in straight microchannels where we studied the spatio‐temporal evolution of domain distribution. The analysis of hundreds of confined GUVs allowed us to establish the probability of having the minority phase within the prescribed free regions. This resulted in the formation of polarized GUVs having one or two Lo or Ld poles at its extremities, surrounding a confined body mainly composed of the other phase. Finally, other confinement geometries allowed us to direct the distribution of lipid domains into 3 or 4 well‐positioned poles.

## Results and Discussion

### Concept

**Figure 1:**
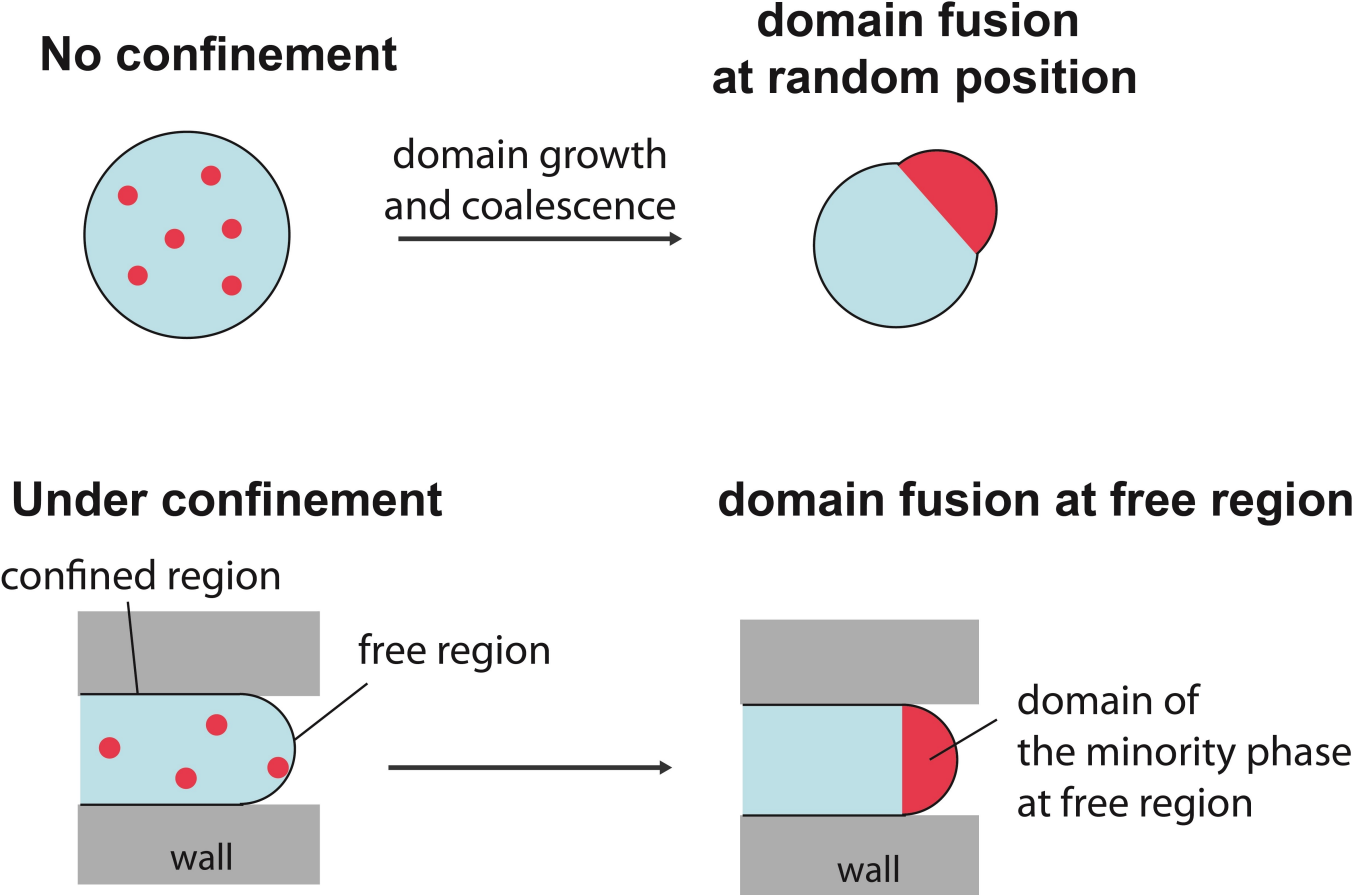
Physical confinement of a phase‐separated Giant Unilamellar Vesicle (GUV) directs lipid domain localization. Without confinement (*top*), domains of the minority phase fuse to grow at random positions, and may bulge out (or bud), to reduce the line energy between co‐existing phases, leading to a multicomponent membrane without control over spatial domain distribution. In contrast, we envisage that, under physical confinement in a microchannel (*bottom*), domains would preferably accumulate and fuse at the free curved regions to minimize line energy, resulting in a domain localization and polarization at this specific position.

It has been theoretically and experimentally shown that the evolution of micro‐scale domains in Lo/Ld phase‐separated GUVs is mainly controlled by line tension, resulting in particular in domain coalescence, growth and budding[^12^, ^19^, ^20^] (Figure 1, *top*). Based on that knowledge, we attempted to control the domain localization, growth, and coalescence by confining individual GUVs in microchannels to create free curved regions at specific positions (Figure 1, *bottom*).

We envisaged that the minority‐phase domains would preferably localize and fuse at the free regions to decrease line energy. A first simple geometric argument in favor of this hypothesis consists in considering the situation of a minority phase forming a single domain with a constant area smaller than that of the free region: its contact line length, and so its energy, would be at minimum in the spherical cap of the free curved region. Moreover, confined GUVs are under tension without much excess area, limiting the possibility of bulging out, especially in the parts in close contact with the channel walls. Bulging is thus expected to have only a minor effect in our system but, should it occur to further decrease the contact line energy, it would also favorably occur in the free curved region. We thus expected accumulation and fusion of the minority‐phase domains at the free curved region to decrease line energy, resulting hypothetically in a single domain forming a pole at each free extremity of the confined GUV (Figure 1, *bottom*). We devised a microfluidic device enabling such a GUV confinement and investigated this concept in detail.

### Membrane Phase Separation in the Confined State

**Figure 2:**
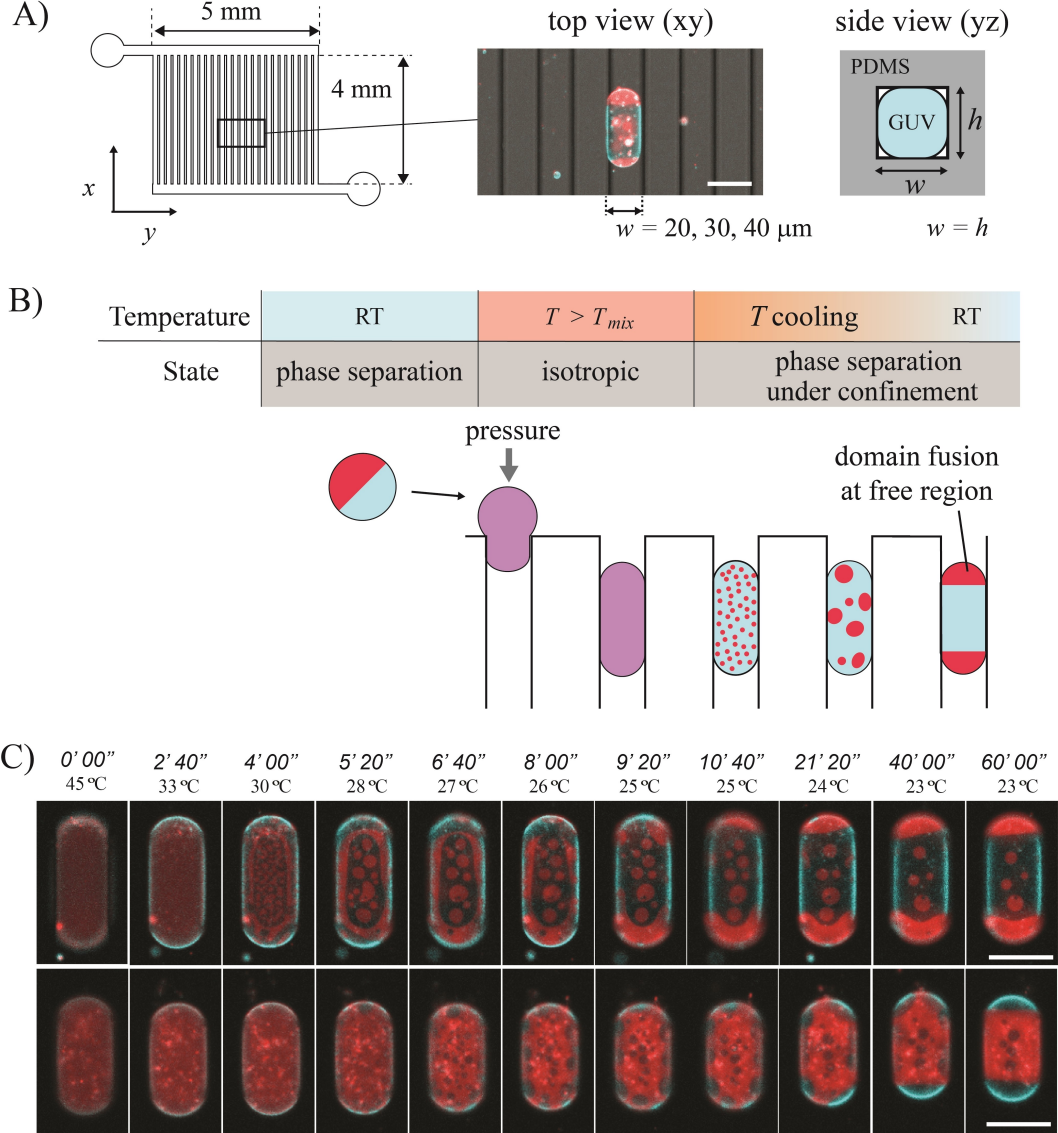
GUVs are confined in a microfluidic device where they phase‐separate, allowing the observation and control over lipid domain distribution. A) Microfluidic channel design (*left* and *right*) and merged fluorescence‐transmission microscopy image (*middle*) of a confined GUV inside a straight channel with a width *w* equaled to its height *h*. B) Experiment principle: GUVs are heated above the mixing temperature (*T*_mix_) prior to microchannel confinement and cooling down to induce phase separation in the confined state. C) Time‐lapse fluorescence microscopy images of confined GUVs in a straight channel (*h*=*w*=40 μm) with a lipid composition DPPC/DOPC/Chol=45/25/30 mol % (*top*, Lo‐rich) and DPPC/DOPC/Chol=40/35/25 mol % (*bottom*, Ld‐rich). The time and the temperature in the channel are indicated above the images. In (A) and (C), red and blue signals correspond to Ld and Lo phase, respectively. All scale bars are 50 μm.

To get GUVs with phase‐separated membranes, we chose the ternary lipid system composed of 1,2‐dipalmitoyl‐*sn*‐glycero‐3‐phosphocholine (DPPC), 1,2‐dioleoyl‐*sn*‐glycero‐3‐phosphocholine (DOPC) and cholesterol, which is known to phase‐separate in liquid‐ordered (Lo) and liquid‐disordered (Ld) phases at room temperature for a range of specific compositions.^[13]^ The confinement was first achieved in parallelly arranged straight microfluidic channels enabling the capture of multiple GUVs in different lanes simultaneously (Figure 2A, Figure S1). GUVs with a typical diameter around 60 μm were confined in channels with a smaller square‐shaped constriction (*h*=*w*=20, 30, or 40 μm, where *h* and *w* denote the designed channel height and width, respectively; see Table S1 for actual values). It allowed us to confine and deform GUVs into rod‐shaped morphologies (Figure 2A, *middle*) where the middle part of each confined GUV was in contact with the flat microchannel walls. In contrast, its extremities constituted free curved regions where the localization, growth and coalescence of lipid domains would thus be favored. GUVs were produced by electro‐formation^[21]^ and introduced in the microfluidic device previously coated with β‐Casein to limit membrane adhesion.^[22]^ The device was heated to 50 °C, i.e., above the mixing temperature (*T_mix_*), to obtain an isotropic membrane^[13]^ and render GUVs more flexible by the increase of membrane surface area as well as by the decrease of their bending rigidity.[^12^, ^23^] Applying a small pressure allowed us to introduce the isotropic GUVs in the confinement channels (Figure 2B). Once the GUVs were confined, the flow inside channels was stopped (Figure S1) to avoid any hydrodynamic effects on domain formation and redistribution. The system was then let to cool down to room temperature (Figure S2) and below *T_mix_*, triggering phase separation in the confined GUVs (Figure 2B). The domain distribution was observed by fluorescence microscopy (Figure 2C) using a small fraction (0.1 mol %) of fluorescently‐labeled phospholipid Rhodamine‐DHPE, which specifically accumulated in the Ld phase (displayed in red), and NBD‐PE, which distributed in both phases thus allowing to visualize the Lo phase in a separate fluorescence channel (displayed in blue).^[24]^ We first used a membrane composition DPPC/DOPC/Chol=45/25/30 mol % leading to a Lo‐rich membrane coexisting with a Ld phase in minority. Notably, time‐lapse observation revealed that the Ld domains progressively accumulated at the free curved region and fused to decrease line energy, first at the “corners” regions (where the membrane became detached from the channel wall) prior to forming a single domain at each tip of the confined GUV (Figure 2C *top*, Movie S1). Close‐up observation revealed that domains tended to move along the channel edges and towards the free curved region (Movie S2). In the confined region, the GUV body is not in strict contact with the surrounding channel walls, especially at the channel edges where the membrane can be slightly deformed toward the free region where it has its maximal curvature. This can create a curvature gradient, which might be at the origin of the domain transport mechanism toward the free curved region. When the same experiment was performed with a composition DPPC/DOPC/Chol=40/35/25 mol %, some GUVs also phase‐separated but with a Ld‐rich membrane (Figure 2C *bottom*, Movies S3–S4). Interestingly, despite a different lipid composition, it was again the phase in minority, in this case Lo, that accumulated and grew at the free regions to form a single domain at each GUV extremity.

### Systematic Localization and Growth of Minority‐Phase Domains at the Free Regions

**Figure 3:**
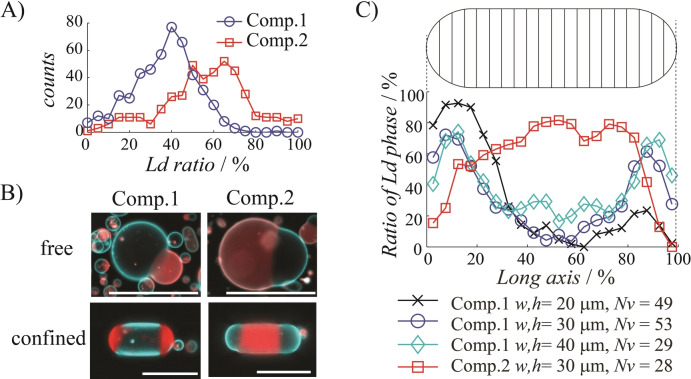
Confining GUVs in straight microchannels directs the growth of minority‐phase lipid domains at the GUV extremities (free regions). A) Distributions of Ld ratio in GUVs with lipid compositions referred to as *Comp.1* (DPPC/DOPC/Chol=45/25/30 mol %) and *Comp.2* (DPPC/DOPC/Chol=30/45/25 mol %). The number of analyzed vesicles was *N*_v_=476 (*Comp.1*) and *N*_v_=428 (*Comp.2*). B) Representative fluorescence microscopy images of Lo‐rich GUVs (*Comp.1*, *left*) and Ld‐rich GUVs (*Comp.2*, *right*), free in solution (*top*) or confined in a straight microchannel (*h*=*w*=30 μm, *bottom*). Red and blue signals correspond to Ld and Lo phase, respectively. C) Localization of Ld phase along confined GUVs. According to the total fluorescence, GUVs were divided into 20 segments (Top) and the projected area fraction of Ld phase (red fluorescence) was measured in each domain. Median values of the ratio of Ld phase along the vesicle length were plotted for different lipid compositions and channel sizes (*Bottom*). The number of analyzed vesicles *N_v_* is indicated below the graph. All scale bars are 50 μm.

To quantify the domain localization phenomenon, we selected two specific lipid compositions leading to different Lo/Ld ratios, referred to as *Comp.1* (DPPC/DOPC/Chol=45/25/30 mol %) and *Comp.2* (DPPC/DOPC/Chol=30/45/25 mol %). Using fluorescence microscopy images, we first measured the projected area fractions of Lo and Ld phases in hundreds of GUVs before confinement and established how GUVs were distributed according to their Ld fraction (Figure 3A). As expected, we found that the small fraction of unsaturated DOPC lipid in *Comp.1* led to a majority of Lo‐rich GUVs, while the DOPC‐rich *Comp.2* resulted in a majority of Ld‐rich GUVs. Without confinement, this phase separation was accompanied by domain coalescence and budding of the phase in minority, i.e., Ld and Lo for *Comp.1* and *Comp.2*, respectively (Figure 3B, *top*). Interestingly, when phase separation occurred in the confined state, we observed that lipid domains corresponding to the minority phase were mainly localized in the free curved regions resulting in rod‐like shaped GUVs with those domains at their tips (Figure 3B, *bottom*), in agreement with the previous time‐lapse observation (Figure 2C). Hence, with *Comp.1* (resp. *Comp.2*), Lo‐rich (resp. Ld‐rich) GUVs had Ld (resp. Lo) domains localized at their tips. We thus obtained the same patterning (minority phase at tips and majority phase in between) with opposite distribution of Lo and Ld phase depending on the initial lipid composition. To characterize the extent of domain localization achieved with this method, we established the distribution of Ld phase, as the projected red fluorescence on the microscopy images, along a normalized longitudinal axis of each confined GUV divided into 20 segments (Figure 3C, *top*). We repeated this analysis on a large number of confined GUVs (Figure S3) and plotted the median value of Ld ratio along this axis for different compositions and geometries of confinement (Figure 3C, *bottom*). This analysis demonstrates a strong and systematic domain localization for both compositions, with Ld domains localizing at the tip (free region) of Lo‐rich GUVs or in the middle body (confined part) of Ld‐rich GUVs. The decrease in the Ld ratio observed in the first and last 5 % of the long axis of the confined Lo‐rich GUVs was attributed to the small fraction of Lo phase that could be trapped there and which was overestimated by our segmentation analysis in this membrane curved region. Since complete switching of the phase localizing at the GUV free region was possible, the localization did not depend on the chemical nature of lipids nor on the type of phase, but was instead directed by the Lo/Ld phase ratio. For *Comp.1*, we also obtained similar distributions regardless of the width and height of the confining channel (Figure 3C, *bottom*). This is attributed to a too low curvature at the free region to induce the type of domain sorting that could be observed with highly curved membranes.^[25]^ It also highlights the robustness of the approach to direct domain localization. All these results show that the minority‐phase domains reproducibly accumulated, coalesced and grew at the free regions of the confined GUVs, regardless of the lipid nature and degree of confinement. This process is attributed to the minimization of line energy through domain accumulation and fusion in the free curved regions.

### Polarization Control

**Figure 4:**
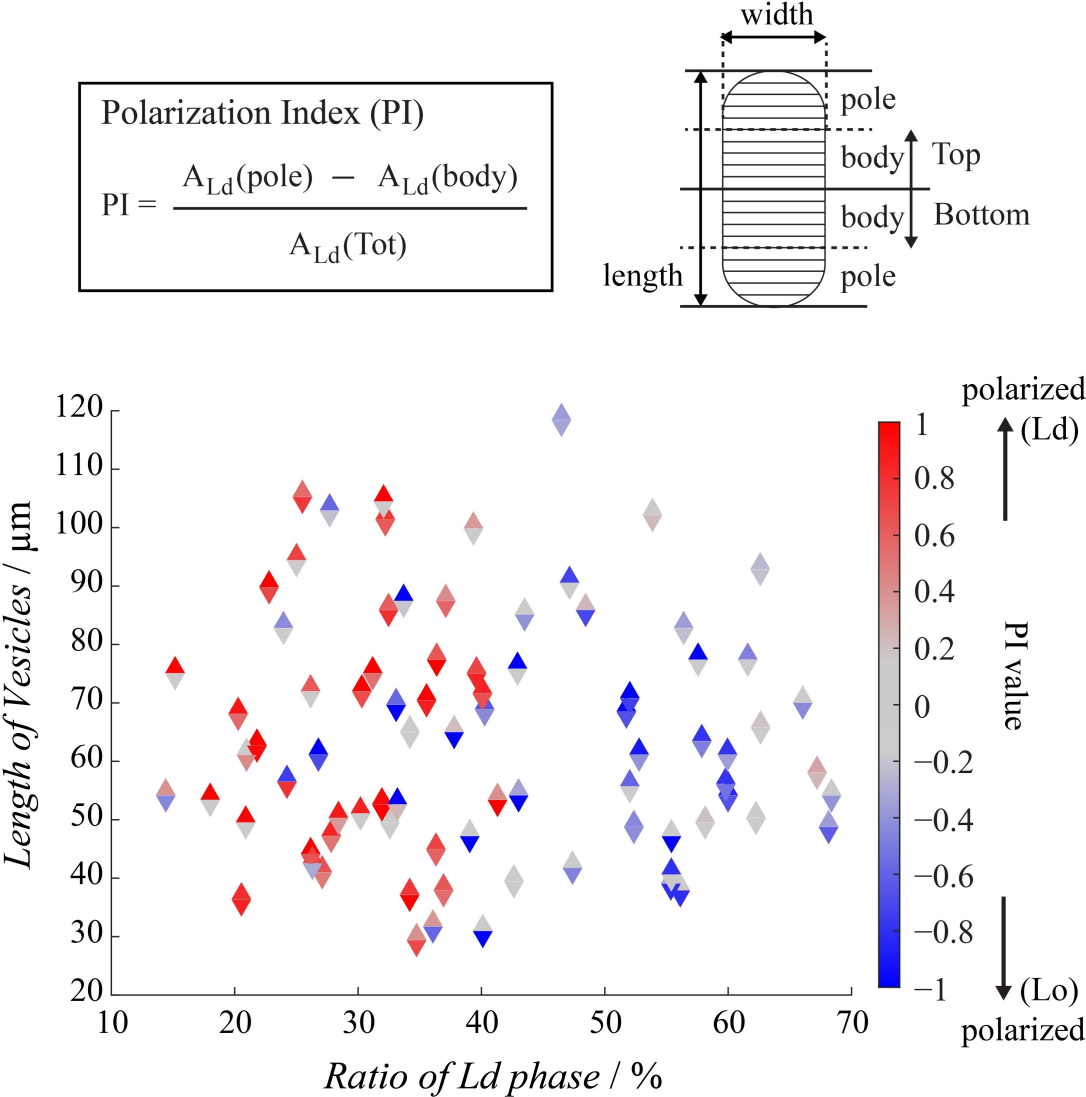
Polarization occurrence in confined GUVs. Polarization index (*PI*=(*A_Ld_(Pole)*−*A_Ld_(Body)*)/*A_Ld_(Tot)* where *A_Ld_(Pole)*, *A_Ld_(Body)* and *A_Ld_(Tot)* represent the projected area occupied by the Ld phase in the pole, body and entire half of the vesicle, respectively), as a function of the length (vertical axis) and Ld ratio (horizontal axis) of GUVs confined in straight channels with *h*=*w*=30 μm. Triangles pointing up and down represent the top and bottom part of each analyzed GUV, respectively. The colors indicate the PI value, which ranges from −1 (strong polarization in Lo phase) to 0 (no polarization) to +1 (strong polarization in Ld phase). Corresponding GUV images and actual PI values are provided in Figure S4 and Table S2.

The domain localization at both ends of the confined GUVs is reminiscent of the polarization that can be observed in some cellular processes. To further quantify this analogy, we developed an image analysis process where each confined GUV detected by fluorescence microscopy was arbitrarily oriented in a top/bottom fashion and segmented into 4 areas of equal length along the longitudinal axis, enabling the definition of a “pole” and a “body” area on each half of the GUV (Figure 4, *top right*).

For each “top” and “bottom” part, we introduced an adimensional number referred to as “polarization index”, *PI*=(*A_Ld_(Pole)*−*A_Ld_(Body))*/*A_Ld_(Tot)*, where *A_Ld_(Pole)*, *A_Ld_(Body)* and *A_Ld_(Tot)* were the projected area occupied by the Ld phase in the pole, body and entire half of the vesicle, respectively (Figure 4, *top left*). This allowed us to systematically categorize the situation of each “top” and “bottom” part of the confined vesicles ranging from a strong polarization of Lo domain (*PI*→−1), to a weak or the absence of polarization (*PI*≈0) to a strong polarization in Ld domain (*PI*→+1). Figure 4, Figure S4 and Table S2 show the resulting polarization diagram where *PI* values for a large number of GUVs confined in straight channels with *w*=*h*=30 μm were plotted in color scale for both the “top” (triangles pointing up) and the “bottom” (triangles pointing down) parts, as a function of the fraction of Ld phase and length of each confined GUV. First, this analysis confirmed the predominant role of the Lo/Ld phase ratio in determining the phase localization. When a strong mono‐ or bi‐polarization occurred (high absolute value of *PI*), it was most of the time corresponding the phase in minority, in agreement with our previous observation. Interestingly, the strongest polarization effects were observed when the minority phase was not in a too small proportion. For instance, vesicles with two strongly polarized parts were mainly observed for Ld≥20 mol % (Ld polarization) and Lo≥40 mol % (Lo polarization). We can explain this effect by the fact that, when a phase is in a very small minority, the low number of initial domains reduced their probability to fuse into a single large domain. In that case, a small pole with the minority phase could be formed at the GUV tip, but some minority phase domains also remained in the body region, decreasing *de facto* the PI absolute value. We also found that the aspect ratio of the confined GUV did not have a strong effect in the occurrence of polarization, except that highly elongated vesicles tended to have a lower *PI* absolute value. This was attributed to a fraction of the minority phase domains remaining in the elongated body region, due to a saturation of the free region and/or by kinetic trapping of the domains that were too far from the free curved region. Finally we found that, under conditions favoring polarization, GUVs were observed with two well‐defined poles in many cases while a strict minimization of line energy would favor the formation of a single pole. We attribute the occurrence of such a bipolarization to the kinetic trapping of the minority phase domains when they entered in any of the two free curved regions imposed by the geometry. Confining GUVs in straight microchannels is thus a way to create polarized cell‐mimicking compartments with partial yet efficient control over the nature and position of the pole(s).

### Concept Generalization and Multipolar GUV Formation

**Figure 5:**
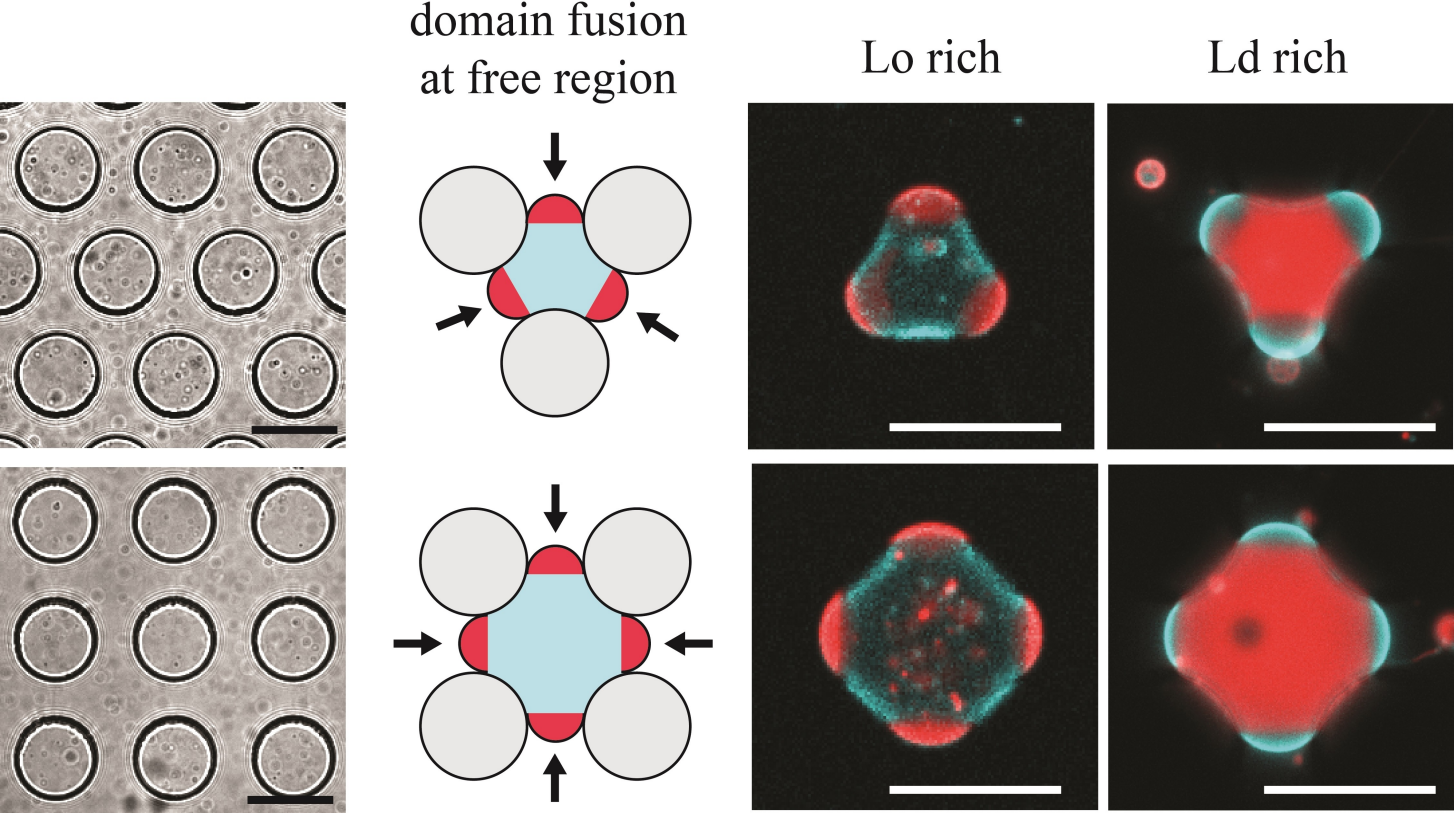
Controlled generation of multipolar GUVs using multiple free regions for domain fusion and localization. Transmission microscopy images of the pillar arrays used to confine the GUVs (*left*). Schematic illustrations (*middle*) of domain positioning (*arrows*) and fluorescence microscopy images (*right*) of GUVs confined between pillars. Lo‐rich GUV with *Comp.1* and Ld‐rich GUV with *Comp.2* were both imaged 60 min after cooling down. Red and blue signals correspond to the region of Ld phase and Lo phase, respectively. All scale bars are 50 μm.

Our understanding of domain positioning was finally applied to control more complex patterning of domain localization. We designed microfluidic chambers with a height *h*=30 μm and periodically spaced 50 μm‐diameter pillars distributed in a hexagonal or squared fashion (Figure 5, *left*) and applied a similar procedure of GUV confinement as with straight channels. Vesicles trapped between the pillars adopted the local geometry and displayed either 3 or 4 free regions for domain fusion according to the pillar arrangement (Figure 5, *middle*). As expected, the free regions defined by the area between the pillars dictated the localization of the minority phase, leading to controlled formation of tripolar and quadripolar GUVs, with well‐positioned poles of Ld (*Comp.1*, Lo‐rich) or Lo (*Comp.2*, Ld‐rich) domains (Figure 5, *right*, Movies S5–S6). With such a confinement geometry, many GUVs were trapped in parallel (Figure S5). Interestingly, all GUVs with a diameter sufficiently large to be deformed by the pillars displayed highly reproducible pole distribution with systematic localization of the minority phase domain in the free regions. In contrast, smaller GUVs were not deformed and displayed random numbers and positions of the poles, confirming the role of confinement to direct the spatial distribution of lipid domains. The mechanism of domain sorting in GUVs under the confinement between pillars was thus consistent with that in straight channels. It is therefore not limited to a specific geometry and appears instead as a general and robust principle that can be exploited to generate GUVs with lipid domains positioned into arbitrary and user‐defined regions.

## Conclusions

Spatial position of lipid domain of liquid‐ordered/liquid‐disordered (Lo/Ld) phase separation in a giant unilamellar vesicle (GUV) was controlled by physical confinement in microchannels. The confinement allowed to determine free curved regions where the lipid domains of the minority phase, either Ld or Lo, became kinetically trapped and favorably accumulated, fused and grew to create single domains at the prescribed areas. The confinement in straight channels and in between pillars reproducibly led to polarized GUVs from one pole to multi poles, depending on the design of microchannel. The approach enabled multiple GUVs confinement, advantageously giving access to data with statistical relevance. The method is also technologically simple, only requiring single‐layer microfluidic chips that can be purchased or custom‐built through conventional microfabrication procedures, offering both accessibility and versatility. As such, it is readily extendable to other types of geometries to spatially control specific lipid domains, domain‐specific membrane‐associated proteins[^26^, ^27^, ^28^] and/or nucleic acids^[29]^ in a user‐defined fashion. We thus anticipate that it will allow shedding light on dynamic membrane reorganization process in model membrane systems as well as constitute a valuable tool for building synthetic cells with better spatio‐temporal control over their component distribution.

## Funding

This project has received funding from the European Research council ERC under the European Unions “HORIZON EUROPE Research and Innovation Programme (Grant Agreement No 101096956)” (D.B.) and the European Community's Seventh Framework Programme (FP7/2007–2013)/ERC Grant Agreement No. 258782 (D.B.), the French National Research Agency ANR contracts DYOR ANR‐18‐CE06‐0019 (D.B.), and an Overseas Research Fellowship from the Japan Society for the Promotion of Science (K.N.). It has received the support of “Institut Pierre‐Gilles de Gennes” (laboratoire d'excellence) and “Investissements d'avenir” program ANR‐10‐IDEX‐0001‐02 PSL, ANR‐10‐ LABX‐31, and ANR‐10‐EQPX‐34.

## Conflict of Interests

The authors declare no conflict of interest.

1

## Supporting information

As a service to our authors and readers, this journal provides supporting information supplied by the authors. Such materials are peer reviewed and may be re‐organized for online delivery, but are not copy‐edited or typeset. Technical support issues arising from supporting information (other than missing files) should be addressed to the authors.

Supporting Information

Supporting Information

Supporting Information

Supporting Information

Supporting Information

Supporting Information

Supporting Information

## Data Availability

The data that support the findings of this study are available from the corresponding author upon reasonable request.
