# Supplementary material for: Controlled Lipid Domain Positioning and Polarization in Confined Minimal Cell Models
Source: Angew Chem Int Ed Engl. 2025 Jan 7;64(7):e202419529. doi: 10.1002/anie.202419529 (PMC11811682; doi:10.1002/anie.202419529)
Supplement: Supplementary file 1 — Supporting Information [file ANIE-64-e202419529-s003.pdf]

Angewandte Chemie
Eine Zeitschrift der Gesellschaft Deutscher Chemiker

Supporting Information

Controlled Lipid Domain Positioning and Polarization in Confined Minimal Cell Models

*K. Nakazawa, A. Lévrier, S. Rudiuk, A. Yamada, M. Morel, D. Baigl**

Supporting Information for:

Controlled lipid domain positioning and polarization in confined minimal cell models

Koyomi Nakazawa[1], Antoine Lévrier,[1] Sergii Rudiuk[1], Ayako Yamada[1], Mathieu Morel[1], Damien Baigl[1]*

[1]PASTEUR, Department of Chemistry, École Normale Supérieure, PSL University, Sorbonne Université, CNRS, 75005 Paris, France

*correspondence to: damien.baigl@ens.psl.eu

Table of contents:

1. Materials and methods
2. Supplementary Figures S1–S5
3. Supplementary Tables S1–S2
4. Legends of supplementary movies S1–S6
5. Supplementary References

1. Materials and methods

Chemicals

1,2-dipalmitoyl-*sn*-glycero-3-phosphocholine (DPPC), 1,2-dioleoyl-*sn*-glycero-3-phosphocholine (DOPC) and cholesterol (ovine wool, >98%) were purchased from Avanti Polar Lipids, N-(7-nitrobenz-2-oxa-1,3-diazol-4-yl)-1,2-dihexadecanoyl-*sn*-glycero-3-phosphoethanolamine, triethylammonium salt (NBD-PE) and Lissamine™ Rhodamine B 1,2-dihexadecanoyl-*sn*-glycero-3-phosphoethanolamine, triethylammonium salt (Rhodamine-DHPE) were purchased from Thermo Fisher Scientific. Sucrose, D^+ glucose, sodium azide, β-casein from bovine milk, PBS buffer were purchased and Indium tin oxide (ITO)-coated glasses (Slide 25 x 75 x 1.1 mm, surface resistivity 15-25 Ω/sq) were from Sigma-Aldrich.

Giant unilamellar Vesicles (GUVs) preparation

GUVs were prepared by the electroformation method using a custom-made protocol adapted from the historical paper[1]. DPPC, DOPC and cholesterol with desired relative amounts were dissolved in chloroform/methanol 9:1 (v/v%) to a total lipid concentration of 20 mM. NBD-PE and Rhodamine-DHPE were added to the lipid mixture (0.1 mol% for each) in order to visualize Lo and Ld phases, respectively, based on the fact that Rhodamine-DHPE distributes in Ld phase while NBD-PE are in both Ld and Lo phases with preference in Lo phase.[2] This lipid solution was spread on the conductive side of two pre-warmed ITO-coated glass slides (70 ºC, 6 µL lipid deposition for each). A lipid dry film immediately formed on the substrate and the film was further dried under vacuum for 3 h. A chamber made by the two lipid-deposited ITO slides and a 1 mm-thick PDMS spacer was then filled by a 100 mOsm/L sucrose, 5 mM NaN_3 aqueous solution. The chamber was sealed with a PDMS plug, inserted into a 70 ºC dry oven and 600 mV AC voltage at 10 Hz was applied for 3 h.

The resulting GUV solution was pipetted to an Eppendorf tube, kept at 4 ºC and used for experiments within one week.

Microfluidic device fabrication

The microfluidic device was fabricated in polydimethylsiloxane (PDMS) using standard photolithography technique.[3] Briefly, the master mold was produced by spin-coating a desired thickness of negative photoresist (SU8-2025, Microchem) on a silicon wafer and exposing it to UV light through a chromium mask of the desired design. The height of obtained channels measured by mechanical profilometer are listed in **Table S1**. After development of the resist, the master mold was coated with a 100 nm layer of parylene by vapor deposition. PDMS (Sylgard 184, Dow) was poured on the master, degassed and then baked overnight at 72 ºC. PDMS was removed from the master, cut, and inlets were pierced with a 1.5 mm-diameter biopsy punch, then sealed with a glass coverslip by oxygen plasma bonding to form a microfluidic channel. To reduce surface interaction with vesicles, channel was passivated by flowing a PBS solution containing 0.5 wt. % β-casein immediately after bonding and for at least 30 min.[4] The channels were then washed with a 100 mOsm/L sucrose solution containing 5 mM of NaN_3 as preservative.

GUV reshaping in the microfluidic device

Experiments were conducted on an epifluorescence microscope (Observer Z1, Zeiss) equipped with a transparent heating stage (Thermo Plate, Tokai Hit), an EMCCD camera (Evolve, Photometrics) and a Metal Halide light source (HXP 120, Zeiss). GUVs solution was first loaded in a 0.5 mm ID Tygon tubing (Tygon S54HL, Saint-Gobain), which was connected to the inlet of the microfluidic device, and flowed in the microfluidic channel using a pressure controller (MFCS, Fluigent). To

avoid strong shear, a small pressure difference (1-2 mbar) was applied between the inlet and the outlet. The chip was placed in a homemade humidity chamber (**Supplementary Figure S1**) to avoid evaporation through PDMS, and temperature was increased to 50 ºC in order to melt the liquid-ordered phase (T_m= ca. 35～38 ºC, depending on the composition)[5] and to increase membrane deformability so that GUVs entered the smaller channels. The absence of residual flow was ensured by closing tubing with clamps at the inlet and the outlet. The heating stage was then turned off, enabling a progressive cooling from 50 ºC to room temperature. Fluorescence image was acquired after 1 h from the beginning of the cooling ramp. NBD-PE was imaged through a FITC filter set (Exc: 475/40 nm Dc: 500 nm Em: 530/50 nm) and Rhodamine-DHPE through a DsRed filter set (Exc: 545/25 nm Dc: 570 nm Em: 605/70 nm). In order to follow the kinetics of distribution and rearrangement of domains (**Figure 2C**), time-lapse images were taken every 20 or 30 s from the beginning of the cooling ramp for 1 h with autofocus (Definite focus, Zeiss).

Image and data analysis

For **Figures 3, 4** and **Supplementary Figures S3, S4, Table S2**, fluorescence images were treated and analyzed using a home-made routine (Fiji). Briefly, full vesicles were segmented from an overlay of the two fluorescence channels and sliced into 20 regions of equal width. Sizes and area ratios of Rhodamine-DHPE positive pixels (red channel) were calculated for the full vesicle and for each regions. Results of GUV detections (projected area, length) and proportions of Ld domains (area ratio of projected Rhodamine-DHPE) along the confined vesicles were further analyzed and plotted using Matlab.

2. Supplementary Figures

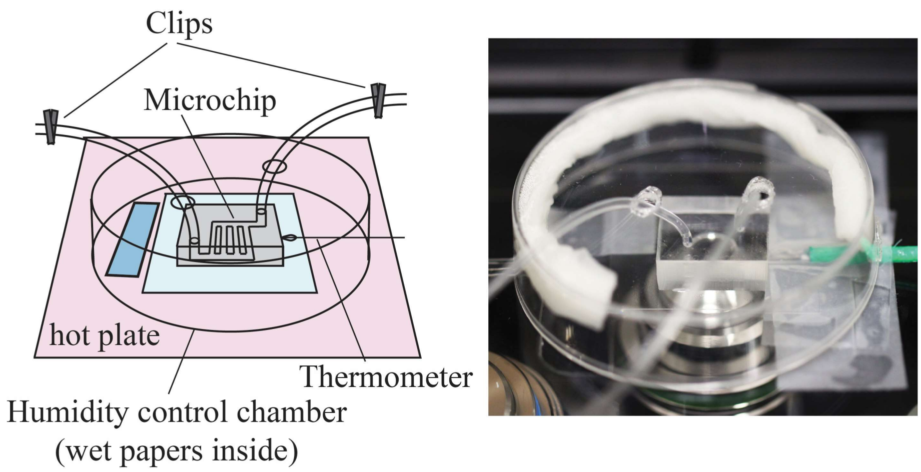

Figure S1. Schematic (left) and picture (right) of the experimental set-up implemented. After GUV introduction in the confinement microchannels, clips were positioned on the inlet and outlet tubes to completely stop the flow.

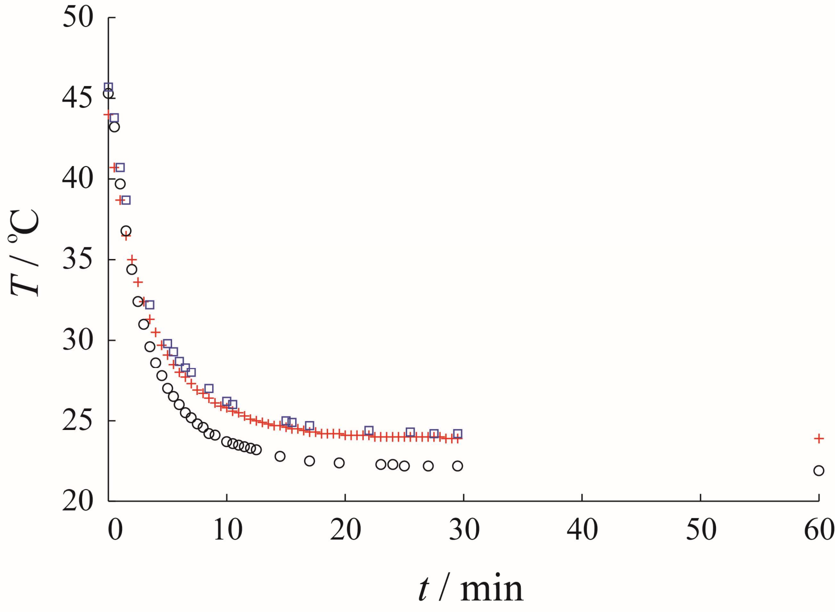

Figure S2 Temperature evolution inside the humidity-controlled observation chamber after heating is stopped. Three identical experiments in different observations were plotted.

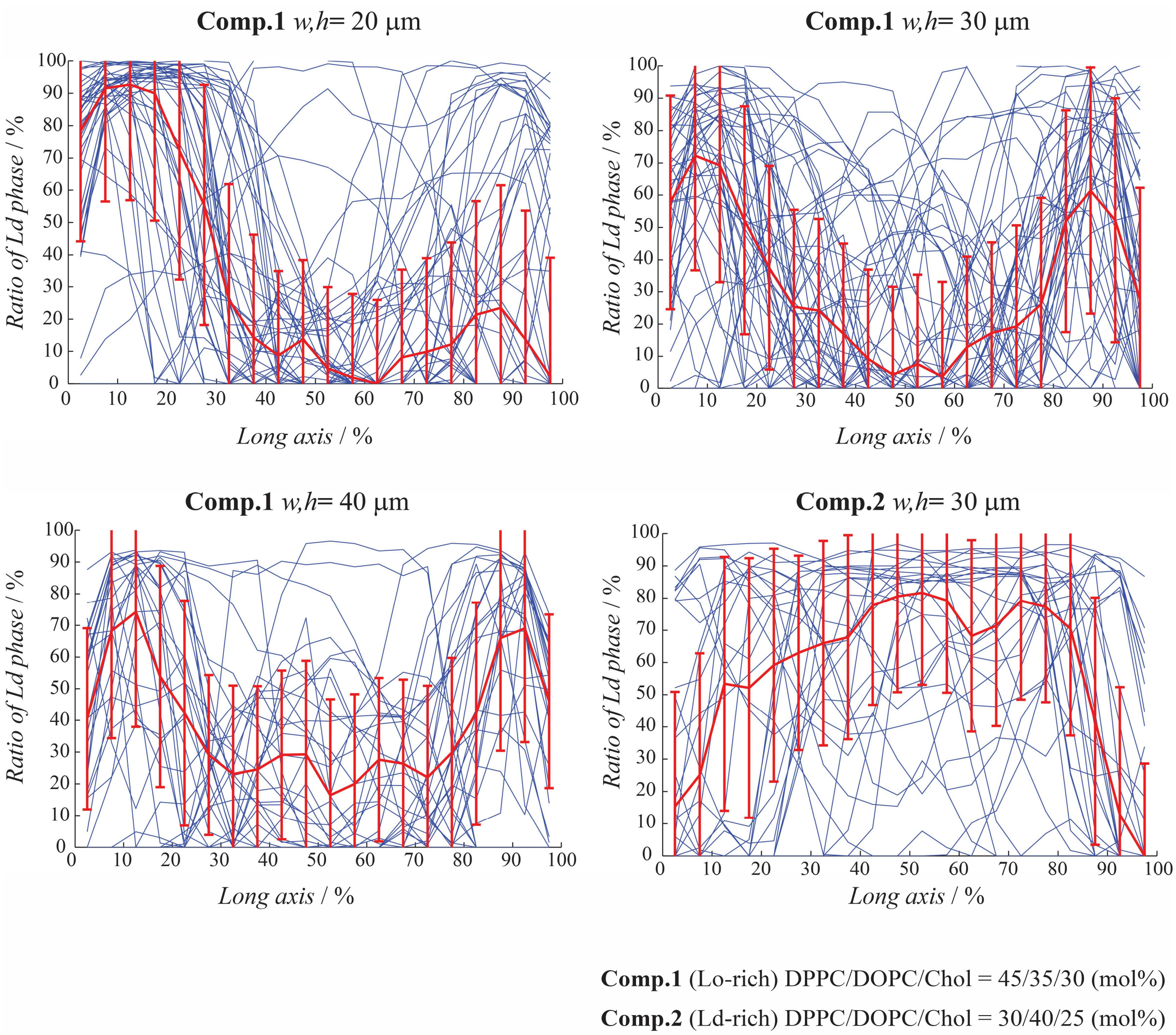

Figure S3. Distribution profiles of the percentage of Ld phase in 20 regions (see Figure 3C in main text) equally and perpendicularly segmented along the normalized long axis of GUV. For each graph, the blue solid lines show the data of single vesicles and the red solid line shows the median value from all vesicles and its standard deviation.

Ld-rich vesicles

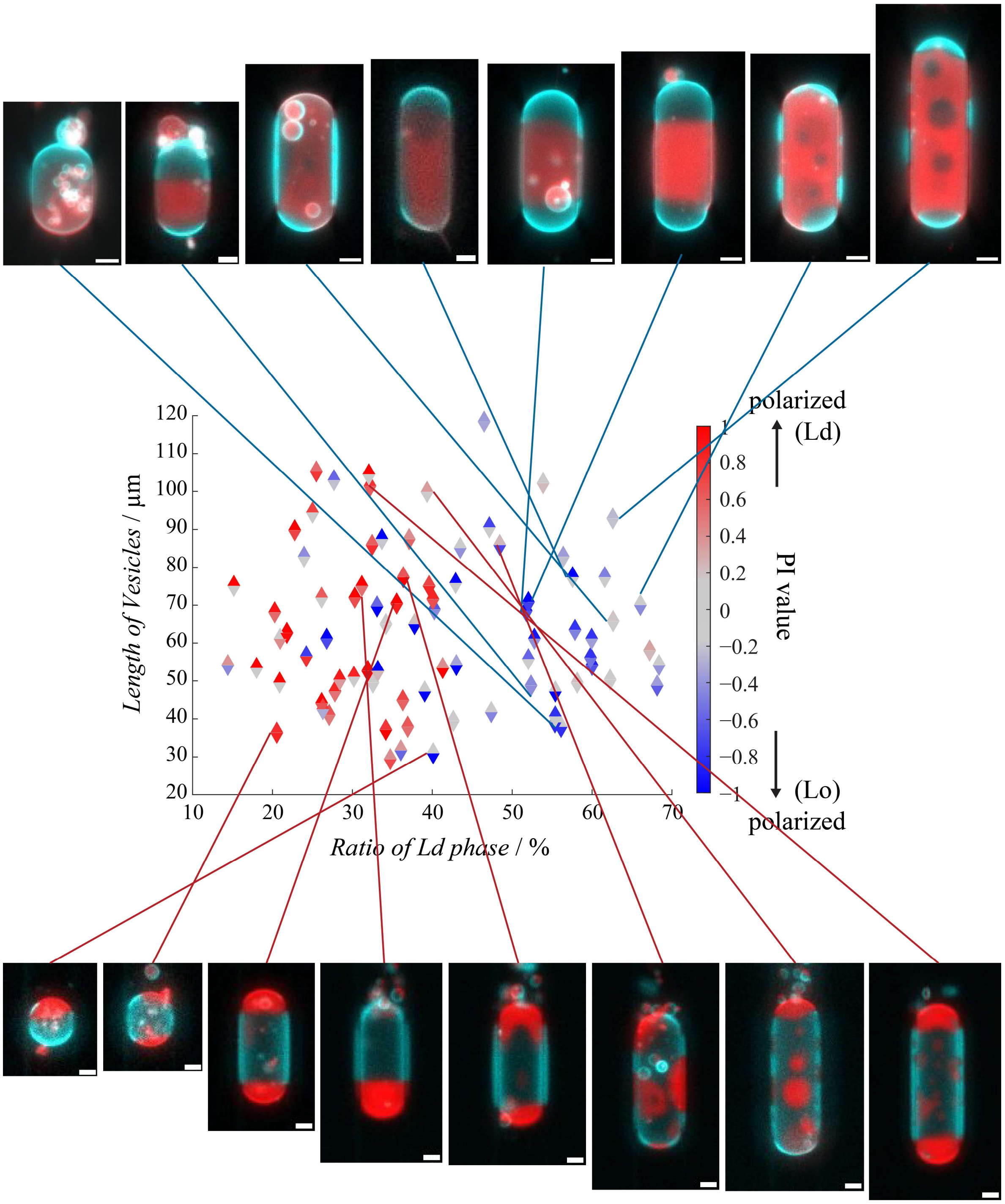

Lo-rich vesicles

Figure S4. Fluorescence microscopy images of some vesicles in the polarization analysis diagram as a function of vesicle length and ratio of Ld phase. Ld and Lo phases are displayed as red and cyan color, respectively. Scale bar: 10 µm.

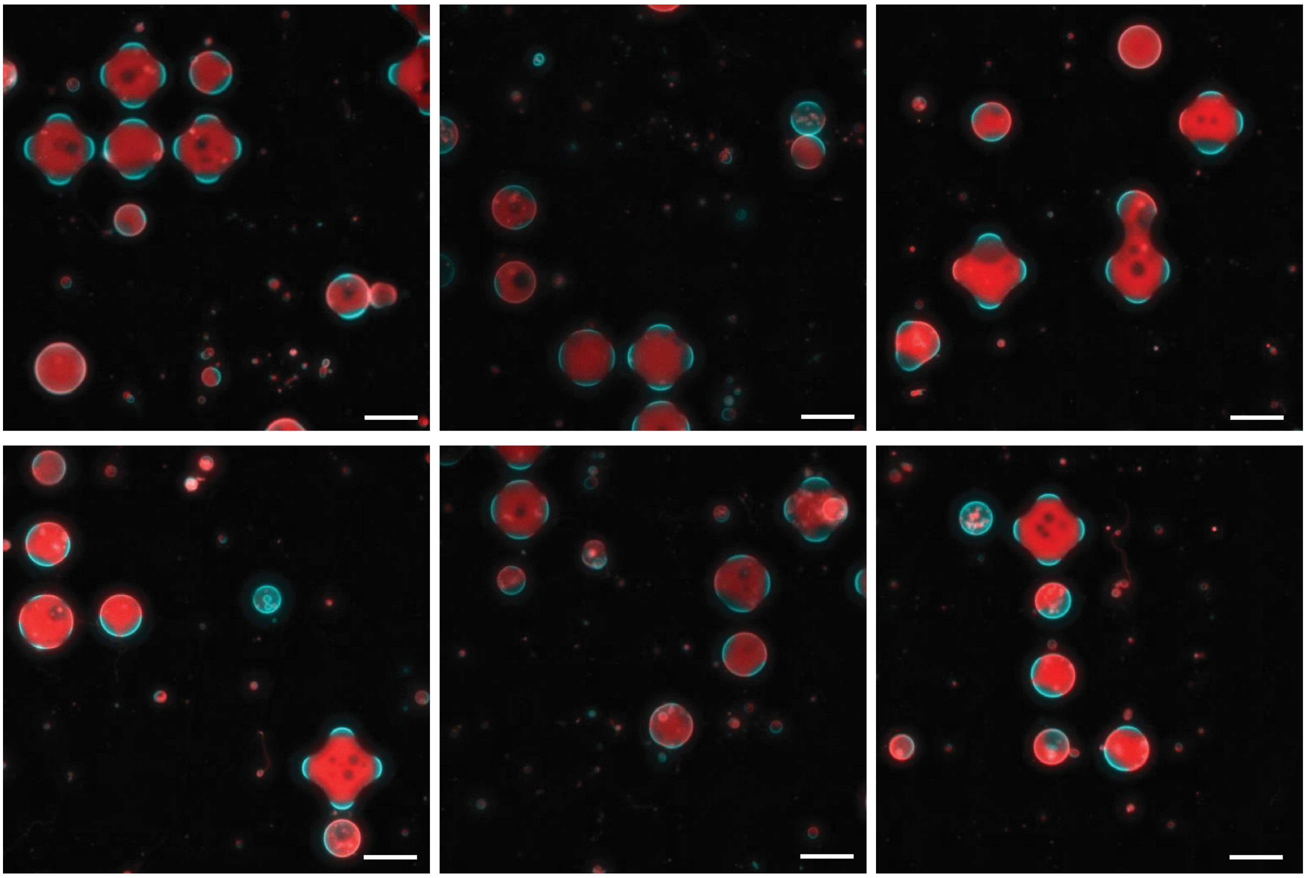

Figure S5. Large-scale fluorescence microscopy images of Ld-rich GUVs (*Comp.2*) confined in the squared array of pillars, imaged 60 min after cooling down. Red and blue signals correspond to the region of Ld phase and Lo phase, respectively. All scale bars are 50 μm.

3. Supplementary Tables

Table S1. Actual dimensions of the straight microchannels compared to the designed ones.

Height (µm)		Width (µm)		Applicable data
Designed	Measured(1)	Designed	Measured(2)	
20	19	20	20	Fig. 2E
30	28.5	30	30	Figs. 2D, 2E, 3
40	42.5	40	40	Fig. 2E
40	36	40	44	Fig. 2F

(1)Measured by mechanical profilometer. (2)Measured from microscopy images.

Table S2. Polarization Index (PI) value of each vesicle plotted in Figure 4

Vesicle	Length (µm)	Ld%	PI value (Top)	PI value (Bottom)
1	77	58	-1.00	-0.14
2	63	58	-0.79	-0.47
3	54	43	-0.33	-1.00
4	49	68	-0.35	-0.62
5	54	60	-0.85	-0.66
6	91	47	-0.72	-0.09
7	83	56	-0.36	-0.23
8	93	63	-0.24	-0.20
9	70	66	-0.16	-0.43
10	71	52	-1.00	-0.74
11	56	60	-0.78	-0.52
12	39	43	0.03	0.11
13	39	55	-0.31	-0.81
14	61	53	-0.90	-0.40
15	50	58	0.20	-0.05
16	61	60	-0.79	-0.35
17	49	52	-0.44	-0.42
18	45	36	0.71	0.59
19	58	67	0.32	0.24
20	56	52	-0.59	-0.17
21	38	56	-0.11	-0.81
22	72	40	0.85	0.67
23	68	52	-1.00	-0.66

24	66	63	0.18	-0.01
25	50	62	0.04	0.03
26	47	55	-0.13	-1.00
27	54	68	0.02	-0.40
28	41	55	-0.80	-0.09
29	77	36	0.64	1.00
30	75	31	1.00	0.54
31	75	40	0.71	0.84
32	70	36	1.00	0.87
33	72	26	0.62	0.00
34	53	18	1.00	0.00
35	47	39	-0.03	-1.00
36	104	32	1.00	-0.10
37	68	20	0.91	0.55
38	100	39	0.36	0.03
39	32	36	0.37	-0.58
40	70	33	-0.57	-1.00
41	63	22	1.00	0.95
42	83	24	-0.42	0.00
43	41	27	0.65	0.49
44	65	38	0.21	-1.00
45	54	14	0.39	-0.44
46	105	25	0.54	0.77
47	94	25	0.72	0.04
48	65	34	-0.08	0.00
49	103	28	-0.58	-0.22
50	61	21	0.00	0.42
51	75	15	0.98	0.00
52	44	26	1.00	0.66
53	36	21	0.81	0.85
54	47	28	0.85	0.52
55	50	28	0.89	0.36
56	31	40	-0.11	-1.00
57	51	30	0.84	0.16
58	38	37	0.63	0.54
59	86	48	0.24	-0.70
60	118	46	-0.36	-0.32

61	102	54	0.10	0.23
62	90	23	1.00	0.70
63	87	37	0.41	0.58
64	87	34	-1.00	-0.19
65	77	62	-0.48	-0.14
66	57	24	-0.75	0.80
67	29	35	0.40	0.68
68	69	40	-0.64	-0.43
69	53	41	0.41	1.00
70	37	34	0.77	1.00
71	72	30	1.00	0.67
72	50	33	-0.15	-0.01
73	76	43	-1.00	-0.15
74	101	32	0.78	0.62
75	86	32	0.61	0.80
76	61	27	-1.00	-0.69
77	43	26	0.74	-0.30
78	53	32	0.97	1.00
79	42	47	0.06	-0.46
80	53	33	-1.00	0.25
81	50	21	0.98	0.00
82	85	43	-0.06	-0.40

4. Legends of supplementary movies

Movie S1

Time-lapse fluorescence microscopy images displaying phase separation and maturation of lipid domains in Lo-rich GUVs confined in straight microchannels ($h = w = 40$ µm). The left GUV corresponds to the one in **Figure 2C *top***. Red and blue signals correspond to Ld and Lo phase, respectively. Images were recorded every 20 s for 1 h. Scale bar: 50 µm.

Movie S2

Time-lapse fluorescence microscopy images of phase separation, coalescence and relocalization of Ld domains (Rhodamine-DHPE) in a Lo-rich GUV confined in a 40 µm-side square channel (same as Movie S1, but closer view on the domain transport and accelerated display). Images were recorded every 20 s for 1 h. Scale bar: 20 µm.

Movie S3

Time-lapse fluorescence microscopy images displaying phase separation and maturation of lipid domains in a Ld-rich GUV confined in straight microchannels ($h = w = 40$ µm). The GUV corresponds to the one in **Figure 2C *bottom***. Red and blue signals correspond to Ld and Lo phase, respectively. Images were recorded every 20 s for 1 h. Scale bar: 50 µm.

Movie S4

Time-lapse fluorescence microscopy images displaying phase separation and maturation of lipid domains in a Ld-rich GUV confined in straight microchannels ($h = w = 40$ µm). Same as Movie S3 but observed in the red channel only (Ld phase). Images were recorded every 20 s for 1 h. Scale bar: 20 µm.

Movie S5

Time-lapse fluorescence microscopy images displaying phase separation and maturation of lipid domains in a Ld-rich GUV confined in the squared array of pillars (same as in **Figure 5 *bottom***). Images were recorded every 30 s for 1 h. Scale bar: 50 µm.

Movie S6

Time-lapse fluorescence microscopy images displaying phase separation and maturation of lipid domains in a Ld-rich GUV confined in the squared array of pillars (same as in **Figure 5 *bottom***). Same

as Movie S5 but observed in the red channel only (Ld phase). Images were recorded every 30 s for 1 h. Scale bar: 50 μm.

5. Supplementary References

[1] M. I. Angelova, D. S. Dimistov, *Faraday Discuss. Chem. Soc.* **1986**, *81*, 303–311.

[2] M. Hishida, R. Yanagisawa, H. Usuda, Y. Yamamura, K. Saito, *Journal of Chemical Physics* **2016**, *144*, 041103.

[3] D. C. Duffy, J. C. McDonald, O. J. A. Schueller, G. M. Whitesides, *Anal Chem* **1998**, *70*, 4974–4984.

[4] A. Yamada, S. Lee, P. Bassereau, C. N. Baroud, *Soft Matter* **2014**, *10*, 5878–5885.

[5] S. L. Veatch, S. L. Keller, *Biophys J* **2003**, *85*, 3074–3083.
